# Downregulation of dual-specificity phosphatase 1 (DUSP1) in post-mortem brain white matter in progressive multiple sclerosis

**DOI:** 10.1007/s10072-026-08944-2

**Published:** 2026-03-12

**Authors:** Martí Oró-Nolla, Melody Cui Sun, Thomas Reilly, Julia Anandarajah, Almudena Otálora-Alcaraz, Lisa Costelloe, Hugh Kearney, Richard Magee, Yvonne M. Nolan, Eric J. Downer

**Affiliations:** 1 Discipline of Physiology, School of Medicine, Trinity Biomedical Sciences Institute, Trinity College Dublin, Dublin 2, Ireland; 2 Department of Neurology, Beaumont Hospital, Dublin, Ireland; 3 MS Unit, Department of Neurology, St. James’s Hospital, Dublin, Ireland; 4 Academic Unit of Neurology, School of Medicine, Trinity College Dublin, Dublin, Ireland; 5 Department of Anatomy and Neuroscience, University College Cork, Cork, Ireland

**Keywords:** DUSP1, Multiple sclerosis, White matter, Microglia/macrophages

## Abstract

**Objective:**

Dual-specificity phosphatase 1 (DUSP1) is a negative regulator of mitogen-activated protein kinase (MAPK) activity, and a player in the control of glial function. Several disease models have identified that the expression of DUSP1 is altered in tissue samples, which may contribute to disease pathogenesis. Using post-mortem brain tissue from a cohort of progressive multiple sclerosis (MS) cases, this study set out to determine if the expression of DUSP1 is altered in the brain in MS.

**Methods:**

We employed the use of a combination of PCR, western immunoblotting and immunohistochemical analysis to profile the expression of DUSP1.

**Results:**

Data presented herein indicate that DUSP1 is significantly downregulated at gene and protein level in MS white matter tissue, when compared to samples from a non-MS control cohort. We also identify that DUSP1 is expressed on Iba1^+^ cells in the brain and that *ITGAM* (CD11b) expression is elevated in white matter in progressive MS.

**Conclusions:**

These findings provide evidence that DUSP1 is dysregulated centrally in CNS white matter in progressive MS.

**Supplementary Information:**

The online version contains supplementary material available at 10.1007/s10072-026-08944-2.

## Introduction

People affected by progressive forms of multiple sclerosis (MS) carry a worse prognosis compared to the relapsing–remitting (RR) course, with primary and secondary progressive forms of MS associated with compartmentalized inflammation, lesion formation and neurodegeneration [1]. In addition, there is a lack of disease-modifying therapies for progressive MS [2]. Microglial cell activation takes place in the cortex in progressive MS [3], and correlates with cortical demyelination [1].

Dual-specificity phosphatase 1 (DUSP1) is a negative regulator of mitogen-activated protein kinase (MAPK) signalling [4]. This is significant given the diverse roles of MAPKs in neural function, with MAPKs linked with inflammatory changes in glia [5]. Studies using the murine model of MS, experimental autoimmune encephalomyelitis (EAE), indicate that DUSP1 deficiency exacerbates disease development [6]. This suggests that lack of DUSP1 can promote inflammation and exacerbate disease pathogenesis, specifically in EAE. There is no evidence to date to indicate that the expression profile of DUSP1 is altered centrally in MS.

The objective of this study was to characterise DUSP1 expression in brain white matter tissue samples from a cohort of progressive MS and non-MS control cases, and determine whether the expression of DUSP1 is altered in MS. We also investigated evidence of inflammatory changes in white matter tissue. The important finding is that there was a significant reduction in DUSP1 expression in white matter from MS cases, when compared to non-MS cases. This may be associated with neuroinflammatory changes that take place in the brain in progressive MS, with further research needed to determine the extent of its contribution to neuroinflammation.

## Materials and methods

Brain samples were provided by the UK MS Tissue Bank and from the Dublin Brain Bank at Beaumont Hospital, Dublin, Ireland. Ethical approval was granted by the Trinity College Dublin School of Medicine Research Ethics Committee (20201004), the Clinical Research Ethics Committee of the Cork Teaching Hospitals (20/2/2013) and the Dublin Brain Bank. Formalin-fixed, paraffin-embedded coronally sliced sections and frozen blocks of coronal slices of cerebrum were obtained from 22 cases for inclusion in the study, including 13 progressive MS cases (*n* = 7 primary progressive (PP) MS and *n* = 6 secondary progressive (SP) MS) and 9 non-MS cases (Table 1). For brain tissue sample selection from non-MS control and MS cases, initial pathological staging and characterization was provided directly by the tissue banks in accordance with published criteria [7], and normal-appearing white matter (NAWM) was used for analysis. Western blot and PCR analysis was performed using frozen white matter tissue blocks of NAWM from non-MS control, PPMS and SPMS cases. Immunohistochemical analysis was performed on 8-µm paraffin-embedded NAWM sections.

**Table 1:** Demographics for brain tissue analysis

Characteristics	Non-MS control	MS	*p* value
Cases, *n*	9	13	
Age at death, years (mean ± SEM)	72.9 ± 5.8	55.5 ± 4.2	0.0213*
Gender (F:M)	3:6	8:5	
Disease duration, years (mean ± SEM)	n/a	20.3 ± 3.8	

MS, Multiple sclerosis; PM, Post-mortem. Disease duration was available for 12 MS cases. Data are expressed as mean ± SEM. *p < 0.05 versus non-MS control. Statistical analysis: Student’s *t*-test

For staining, brain sections containing NAWM were de-paraffinized and re-hydrated prior to heat-mediated antigen retrieval. Sections were blocked in donkey serum (Sigma) and stained with mouse monoclonal Iba1 antibody (1:100; Abcam) and rabbit monoclonal DUSP1 antibody (1:500; Bio-Techne) overnight at 4°C. Sections were probed with donkey anti-mouse Alexa647 and anti-rabbit Alexa488 antibodies (1:1000 in 5% donkey serum; ThermoFisher) for 1 h in the dark. Nuclei were stained with DAPI and sections were mounted in Dako mounting medium.

For western blot analysis punchers were used to extract NAWM from each tissue block. Tissue was homogenised in N-PER Extraction Reagent supplemented with 1% Halt™ protease/phosphatase inhibitors and 1% EDTA (ThermoFisher). Samples were placed on ice for 20 min and centrifuged at 14,000 rpm at 4°C for 20 min. Supernatants were collected and equalized lysates mixed with 2 × Laemmli Sample Buffer (Sigma), heated to 95°C for 5 min and run on 10% acrylamide gels. Proteins were transferred to PVDF, blocked (60 min) and incubated overnight at 4°C with rabbit anti-MKP-1 (1:500; ThermoFisher) and mouse anti-β-actin (1:10,000; ThermoFisher). Membranes were incubated with goat anti-mouse (Li-Cor) or anti-rabbit (Li-Cor) secondary antibodies (1:10,000). Densitometry was performed using ImageJ.

For PCR analysis samples of NAWM were homogenized in RA1 buffer (Macherey–Nagel) supplemented with β-mercaptoethanol (1:100). RNA was extracted using NucleoSpin® RNAII kits (Macherey–Nagel). cDNA synthesis was performed using High Capacity cDNA Kits (Applied Biosystems). Primers used were DUSP1 and integrin-α-M (ITGAM) (Assay no. Hs00610256_g1 and Hs00355885_m1). cDNA was mixed with qPCR™ Mastermix (Applied Biosystems) and the respective gene assay. Eukaryotic 18S rRNA was used as a control and expression was assessed using gene expression assays containing forward/reverse primers and a VIC-labelled MGB Taqman probe (#4319413E; Applied Biosystems). Analysis was performed using the 2^−ΔΔCT^ method.

Data were tested for normality using the Shapiro–Wilk test. If data were normally distributed, parametric testing was used via Student’s *t*-test. If data were not normally distributed, non-parametric testing was employed using the Mann–Whitney U test. Criteria for significance was *p* < 0.05. Data are expressed as means ± standard errors of the mean.

## Results

**Fig. 1:**
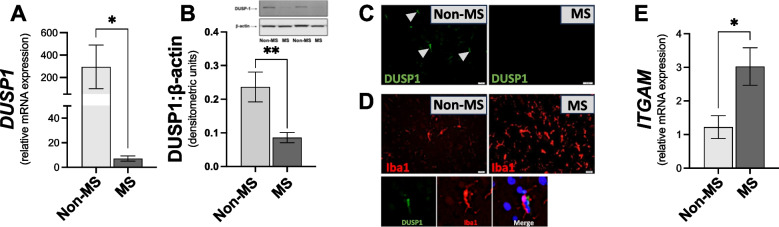
The expression of DUSP1 is reduced in white matter tissue in the cerebrum in progressive MS cases. DUSP1 (**A**) gene and (**B**) protein expression in white matter tissue samples from non-MS control cases and from a cohort of progressive MS. Data are expressed as mean ± SEM from control (*n* = 8–9) and progressive MS (*n* = 12–13) cases. (**C**) Immunofluorescent images demonstrating DUSP1 expression in brain sections. (**D**) Immunofluorescent images demonstrating Iba1 expression, and co-localisation of Iba1/DUSP1 expressing cells in CNS brain sections. (**E**) *ITGAM* expression in brain tissue samples from non-MS control cases (*n* = 5) and progressive MS cases (*n* = 11). Statistical significance was determined by the Student’s *t*-test or Mann–Whitney test. *p < 0.05, **p < 0.01 *versus* non-MS control group

Given the evidence that the expression of DUSP1 is altered in brain tissue in neurological disorders [8], we set out to characterise the expression of DUSP1 gene transcripts and protein in NAWM in the cerebrum in post-mortem samples, and to determine if the expression profile is altered in MS. Data presented in Fig. 1A indicate that the expression of *DUSP1* was reduced in NAWM from MS cases, when compared to samples from non-MS cases (Fig. 1A). In addition, western blot analysis indicates that DUSP1 protein expression was significantly reduced in white matter tissue lysates from progressive MS cases (Fig. 1B). These findings were supported by fluorescence microscopy, with evidence that DUSP1 immunoreactivity was greater in brain tissue from non-MS cases, when compared to progressive MS samples (Fig. 1C). This indicates that the expression of endogenous DUSP1 is reduced centrally in brain NAWM from progressive MS cases.

To determine whether inflammatory changes are taking place within the brain in progressive MS, we investigated the expression of microglia/macrophage specific markers. Using fluorescence microscopy we identified Iba1^+^ cells (microglia/macrophage marker) in non-MS control and MS NAWM, with evidence that Iba1^+^ immunoreactivity was elevated in MS (Fig. 1d). The data also show that DUSP1 colocalized with Iba1^+^ cells (Fig. 1d). Furthermore, *ITGAM* (which encodes for CD11b) expression was significantly increased in NAWM from progressive MS cases, when compared to non-MS control NAWM (Fig. 1e). This suggests that the number (and/or reactivity) of microglia/macrophages is elevated in progressive MS samples.

## Discussion

This study set out to characterise the expression of DUSP1 in the post-mortem brain, and to determine if the expression profile is altered centrally in progressive MS. Changes in the endogenous expression of DUSP1 in the CNS have been shown in other disease states/models [8]. The key finding is that there was a significant reduction in DUSP1 expression in NAWM from MS cases, when compared to NAWM from non-MS cases. To our knowledge this is the first evidence to indicate that the expression of this phosphatase is altered centrally in MS.

DUSP1 is also expressed in immune cells [9], and data presented herein supporting this (Supplemental Fig. 1). We found no difference in *DUSP1* expression between immune cells from individuals with MS and healthy volunteers. This suggests that the dysregulation of DUSP1 in MS is restricted to cells of the CNS, and not immune cells. Importantly, recent data from multiple MS white matter transcriptome datasets did not systematically identify DUSP1 as a significantly deregulated gene in MS white matter [10]. Such differences may reflect variations between sample cohorts and methods used to assess gene signatures.

MS more commonly affects women, and sex-specific responses of DUSP1 [11] to stress/injury have been determined. We found no sex-related differences in *DUSP1* expression in brain white matter samples in the bank of tissue assessed (Supplemental Fig. 2).

DUSP1 is a regulator of a variety of functions, and has been reported to negatively regulate microglial activation [12]. In terms of MS, DUSP1 has been reported in MS brain lesions in microglial cells [13], and our data support this. However, there is no evidence to date to indicate that DUSP1 expression is altered in the brain of individuals with MS. We report that *ITGAM* expression was elevated in MS tissue. The ITGAM gene, which encodes for CD11b, is expressed in macrophages/microglia, and can regulate diverse cell functions in terms of adhesion/migration. Given that *ITGAM* was elevated in progressive MS CNS tissue, our findings suggest that the number (and/or reactivity) of microglia/macrophages is elevated in progressive MS brain samples.

A study limitation is that DUSP1 expression was not characterised in chronic active lesions (CALs), with analysis restricted to NAWM. A further study limitation is that the brain tissue assessed was not region matched between cases.

Overall we have identified a marked difference in the expression of DUSP1 (at gene/protein level) in CNS NAWM in progressive MS. Given that EAE studies indicate that DUSP1 deficiency exacerbates disease development [6], we hypothesize that the changes in DUSP1 expression in the CNS identified herein may contribute to neuroinflammatory changes that take place in MS. This warrants further investigation and will be the focus of future studies. In addition, given the lack of DMTs for progressive MS, targeting DUSP1 regulation centrally may represent a new therapeutic avenue for therapeutics in development for progressive forms of MS.

## Supplementary Information

Below is the link to the electronic supplementary material.

Supplementary file1 (PPTX 113 KB)

## Data Availability

The datasets generated and/or analyzed for the study may be madeavailable from the corresponding author upon reasonable request.
