# Supplementary material for: Downregulation of dual-specificity phosphatase 1 (DUSP1) in post-mortem brain white matter in progressive multiple sclerosis
Source: Neurol Sci. 2026 Mar 12;47(4):338. doi: 10.1007/s10072-026-08944-2 (PMC12979298; doi:10.1007/s10072-026-08944-2)
Supplement: Supplementary file 1 — Supplementary file1 (PPTX 113 KB) [file 10072_2026_8944_MOESM1_ESM.pptx]

## Slide 1
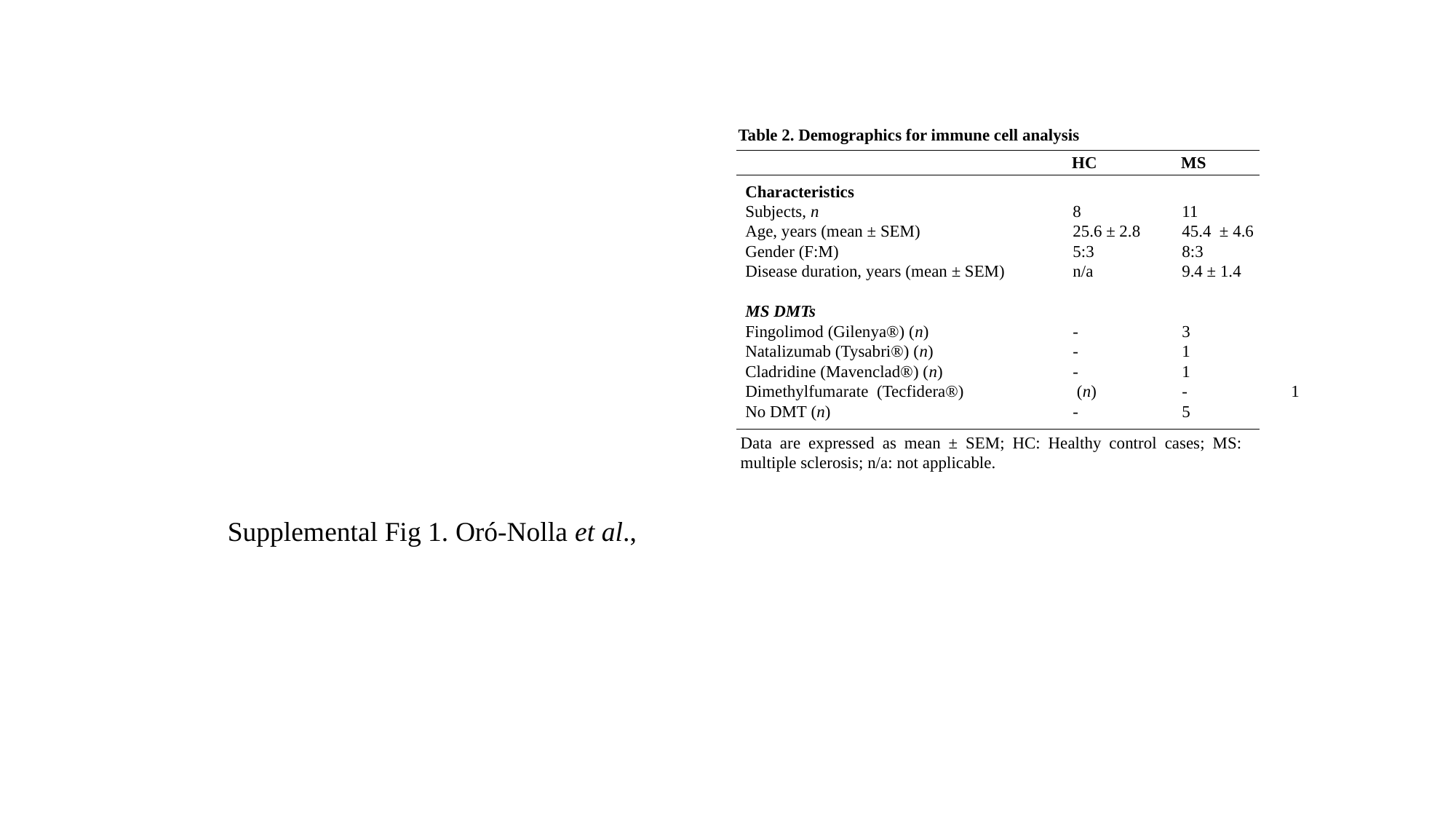

Table 2. Demographics for immune cell analysis
HC 	MS
Characteristics
Subjects, n			8	11
Age, years (mean ± SEM)		25.6 ± 2.8	45.4 ± 4.6
Gender (F:M)			5:3	8:3
Disease duration, years (mean ± SEM) 	n/a	9.4 ± 1.4
MS DMTs
Fingolimod (Gilenya®) (n)		-	3
Natalizumab (Tysabri®) (n) 		-	1
Cladridine (Mavenclad®) (n) 		-	1
Dimethylfumarate (Tecfidera®)	 (n) 	-	1
No DMT (n) 			-	5
Data are expressed as mean ± SEM; HC: Healthy control cases; MS: multiple sclerosis; n/a: not applicable.
Supplemental Fig 1. Oró-Nolla et al.,

## Slide 2
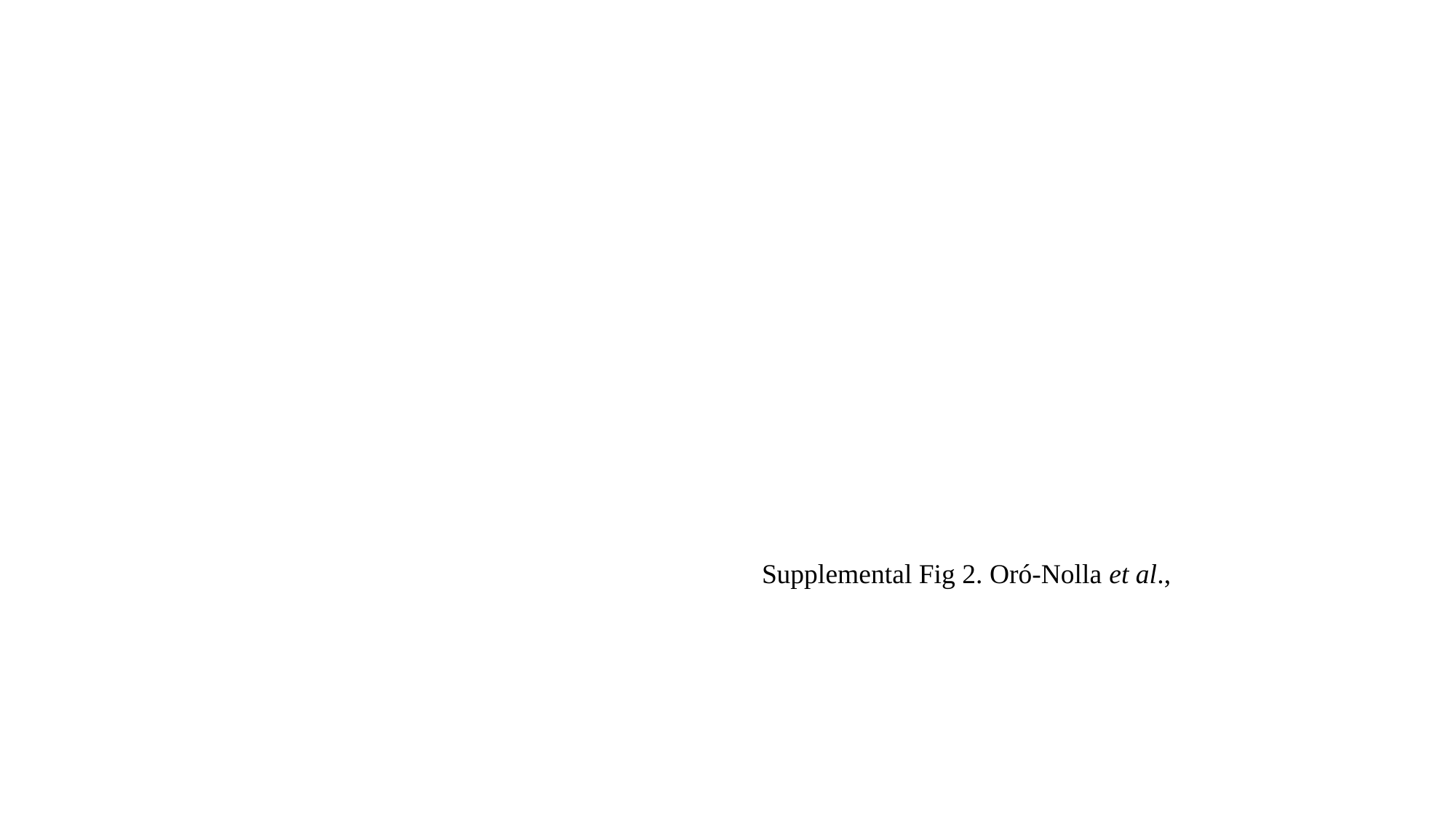

Supplemental Fig 2. Oró-Nolla et al.,
